# Supplementary figures and images for: The Neisseria gonorrhoeae type IV pilus promotes resistance to hydrogen peroxide- and LL-37-mediated killing by modulating the availability of intracellular, labile iron
Source: PLoS Pathog. 2022 Jun 17;18(6):e1010561. doi: 10.1371/journal.ppat.1010561 (PMC9246397; doi:10.1371/journal.ppat.1010561)

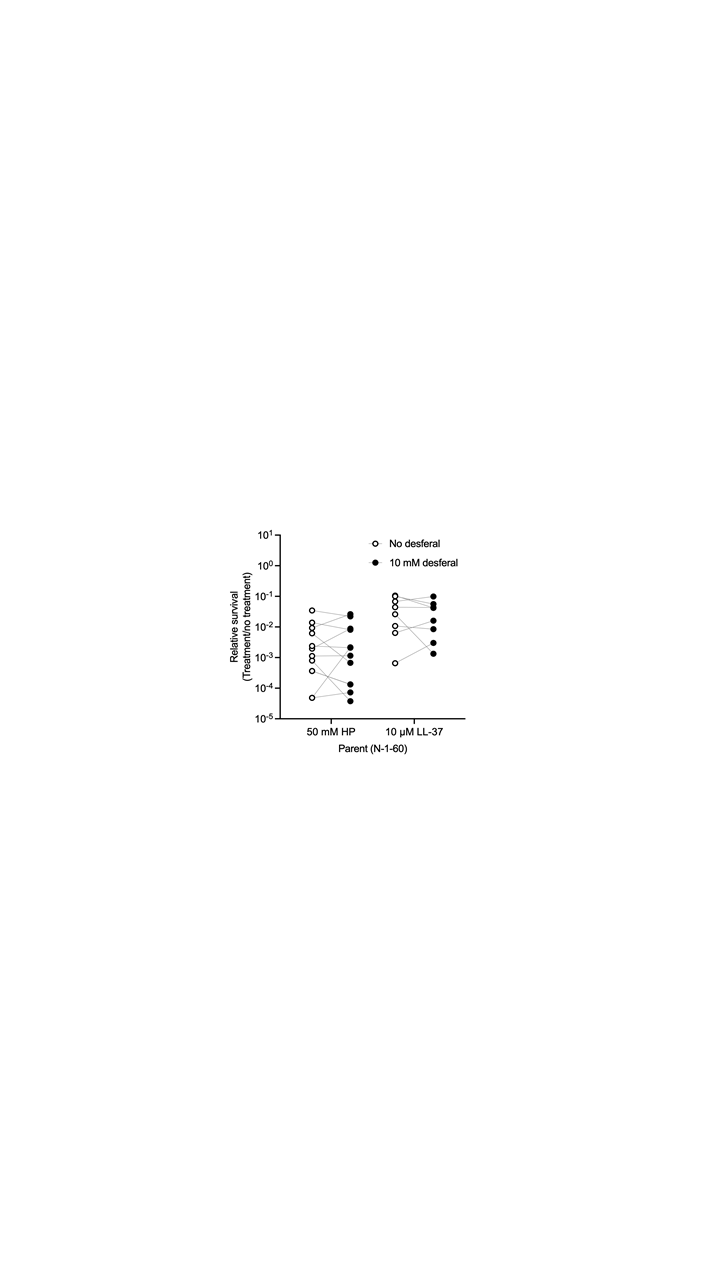

Supplement: S1 Fig — Relative survival of the FA1090 parent strain N-1-60 to hydrogen peroxide or LL-37 after desferal treatment was determined. The lines indicate the paired samples. The average relative survival of the parent to hydrogen peroxide was 0.0059 with 10 mM desferal and 0.0059 without desferal. The average relative survival of the parent to LL-37 was 0.033 with 10 mM desferal and 0.045 without desferal. (TIF) [file ppat.1010561.s001.tif]

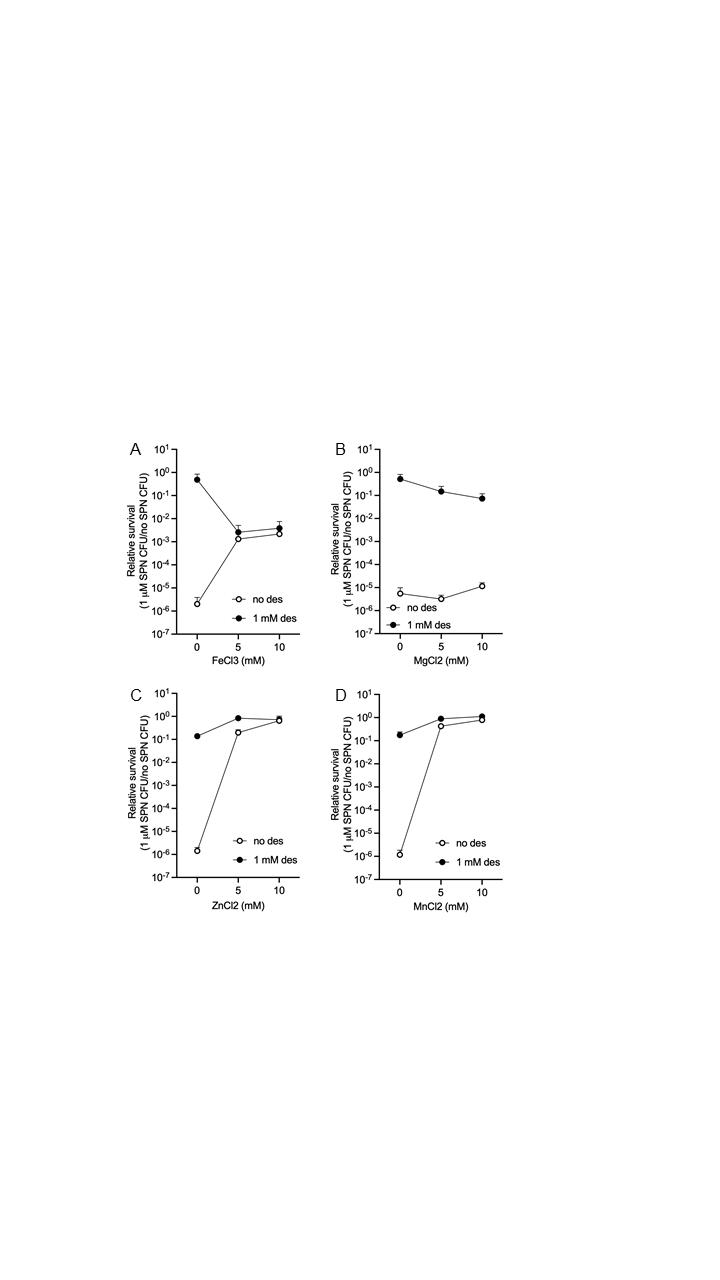

Supplement: S2 Fig — The pilE mutant was treated with either desferal alone or desferal mixed with various concentrations of FeCl3, n = 3 (A), MgCl2, n = 3 (B), ZnCl2 n = 3 (C), or MnCl2 n = 3 (D) before streptonigrin killing. Relative survival was determined by calculating the ratio of streptonigrin resistant colonies to the total number of cells in the reaction. The averages and the standard error of the mean are shown. (TIF) [file ppat.1010561.s002.tif]

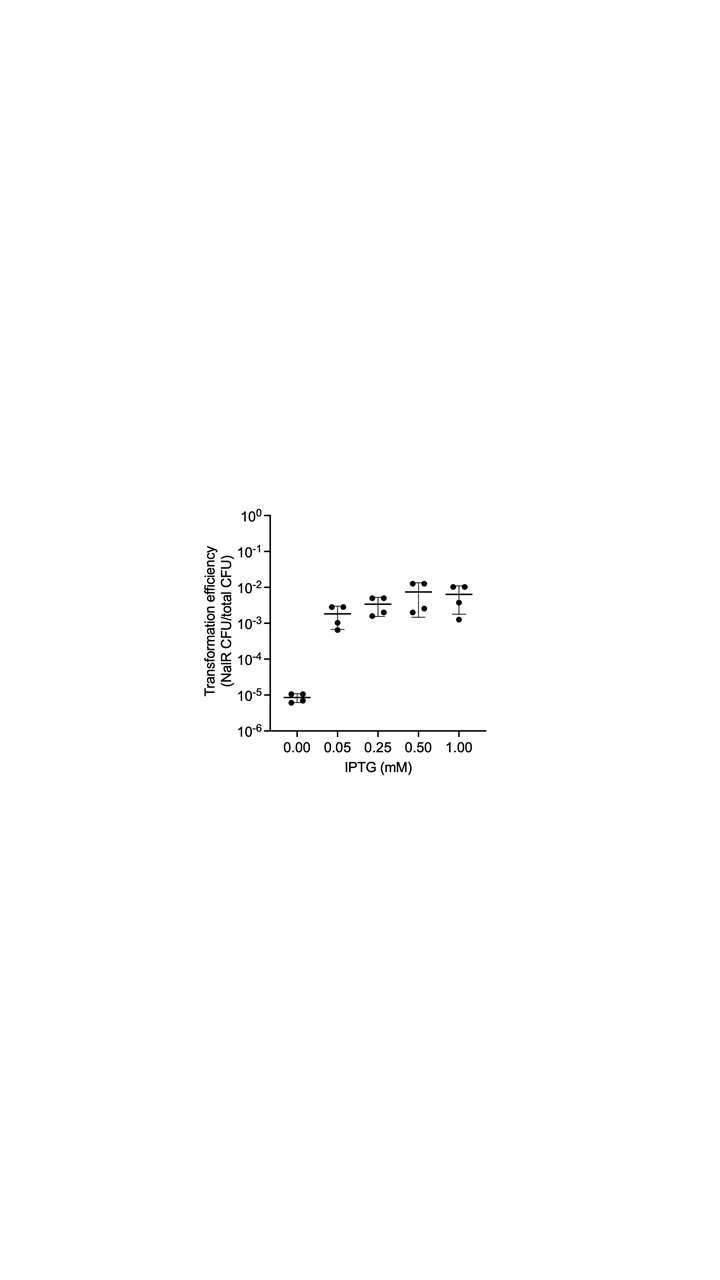

Supplement: S3 Fig — The transformation efficiency of the FA1090 strain carrying an IPTG inducible pilE was determined using pSY6, a plasmid that carries a gyrA point mutation. The number of nalidixic acid-resistant colonies was compared to the total number of colonies in four biological replicates. The average and standard error are shown. (TIF) [file ppat.1010561.s003.tif]

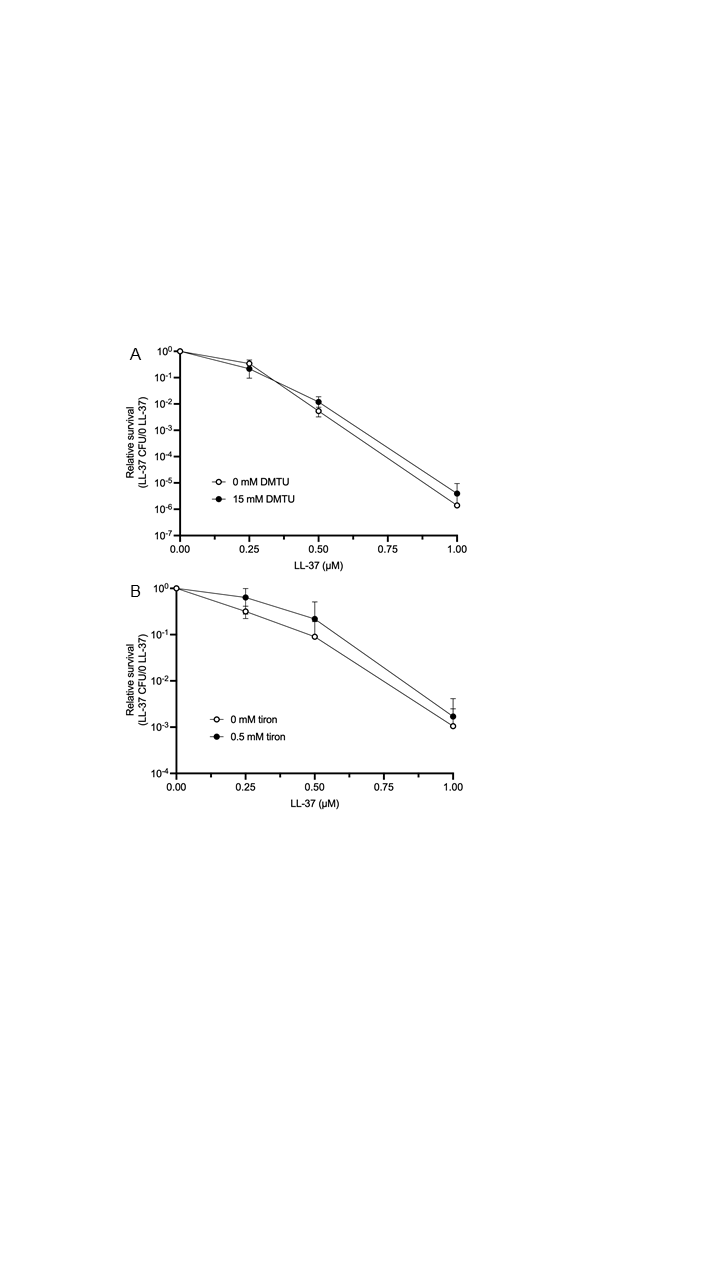

Supplement: S4 Fig — The effect of DMTU (A, n = 3) and tiron (B, n = 4) on LL-37 sensitivity in the pilE mutant. The mean and standard error of the mean are plotted and analyzed by Wilcoxon matched-pairs signed-rank test but are not statistically significant. (TIF) [file ppat.1010561.s004.tif]
