# Supplementary material for: The Neisseria gonorrhoeae type IV pilus promotes resistance to hydrogen peroxide- and LL-37-mediated killing by modulating the availability of intracellular, labile iron
Source: PLoS Pathog. 2022 Jun 17;18(6):e1010561. doi: 10.1371/journal.ppat.1010561 (PMC9246397; doi:10.1371/journal.ppat.1010561)
Supplement: Attachment — Submitted filename: Response to reviewers.docx [file ppat.1010561.s005.docx]

**Response to Editor’s Comments**

The reviewers acknowledged the quality of this work. But they also pointed out that: (i) no clear mechanism is identified as to how pili alter iron homeostasis and (ii) the results could be discussed further. I also encourage the authors to pay particular attention to points 14 and 15 of reviewer #1. These points should be addressed before resubmission.
I apologize for the duration of the review process, which was necessary to produce an effective evaluation of this work.

**We appreciate you getting the reviews back to us as fast as possible and the reviews have been helpful in producing an improved manuscript that we believe is now acceptable for publication in PloS Pathogens. We agree there is no clear mechanism but we have included a thorough discussion and an accompanying model in Figure 5 on the possible mechanism(s) and are investigating between different hypotheses, but these findings will be part of a future report. We have also discussed the results further and explained better our conclusions in various parts of the paper.**

**Response to Reviewer’s Comments (responses in bold type)**

Reviewer #1:

REPORT FOR TRANSMISSION TO AUTHORS

The manuscript by Hu et al addresses the mechanism as to why piliated cells of N. gonorrhoeae are more resistant to oxidative killing by hydrogen peroxide and to killing by the antimicrobial peptide LL-37. The manuscript builds on previous results of the authors showing that: i) the metalloprotease MPG (NGO1686) is highly expressed in response to sublethal levels of hydrogen peroxide, ii) MPG is required for full piliation of gonococcal strains and the latter is required for resistance to hydrogen peroxide and LL-37 killing. N. gonorrhoeae is provided with multiple genetic mechanisms to resist oxidative damage. This mechanistic redundancy highlights the importance of resistance to oxidative killing for pathogenesis. However, very often the mechanism by which these gene products afford resistance to oxidative damage is unknown. Base on this, the theme of the paper is highly relevant in the context of gonococcal pathogenesis.

**We appreciate the reviewer’s view that “the theme of the paper is highly relevant”.**

However, there are some problems, which can be addressed editorially, in the way data is presented and the conclusions drawn from the results (see below). This reviewer believes that although evidence is presented to suggest that the way full piliation protects cells from hydrogen peroxide oxidative damage is by restricting the intracellular pool of labile iron, an additional experiment is required to fully validate the evidence as conclusive (see point 14 below).

**We thank the reviewer for their editorial suggestions and the recommended experiment in point 14. We have attempted to directly measure the labile iron pool and believe that the experiment requires further optimization.**

1. In figure 1C and D the pilE mutant should be presented side-by-side with the wild type, so that one can compare the magnitude by which desferal rescue survival in the mutant and the wild type. Given that hydrogen peroxide requires iron in order to kill cells, addition of an iron chelator (desferal) is expected to protect any type of cell; therefore, the problem here is that without comparing the magnitude of protection between mutant and wild type one cannot conclude whether sensitivity of the mutant is due to iron content.

**We have included these experiments in the modified manuscript as Figure S1.**

2. Similar to point 1 above, in figure 2B the side-by-side comparison with the wild type is not presented. In fact, in line 169 the authors discuss the result of desferal and streptonigrin treatment on the wild type strain, but I could not find the result.

**The data is now included as Figure 2C.**

3. In figure 2C the addition of extra iron seems to increase survival to streptonigrin in the absence of desferal (results discussed in text in line 179-181), while extra iron in the presence of desferal lowered survival (expected). This result seemingly contradicts the logic of figure 2B in which desferal protects mutant cells from streptonigrin killing. The author should explain why extra iron in the absence of desferal seems to increase survival. Perhaps statistical comparisons between the ±iron without desferal conditions and ±iron with desferal conditions are required to understand the significance of this result.

**We included our speculation on why iron increases resistance to streptonigrin (lines 475-478) and included the statistical analysis on streptonigrin-treated cells that were not treated with desferal with and without iron in Figure 2D.**

4. The result describing the survival in the presence of other metals as presented in line 182 is not represented anywhere. PloS Pathogens does not allow “Data not shown”; hence, the authors should show the results in a supplemental figure.

**The data are included in the text (lines 231-245) and Figure S2.**

5. The authors do not explain why the survival ratio (approx. 6E-4 in Fig.3) of the IPTG-inducible pilE mutant without IPTG seems to be significantly lower than the pilE mutant strain (N-1-69) in figure 2B. Perhaps the authors should present results from both strains in the same graph, or comment about this difference.

**The value of commenting on the difference does not seem critical to us. The experiments were performed at different times using different stocks of reagents. We have found that streptonigrin stock shelf life has an effect on killing efficacy. The strains are also not isogenic to one another; therefore, strain construction may have introduced uncharacterized mutations in the genome that could affect sensitivity.**

6. I may have missed it, but in line 218 the authors present figure 3B which is not in the manuscript.

**Thank you. It should have been Figure 4, which we have removed from the revised manuscript.**

7. In figure 4 the authors did not find any statistical differences among different mutations with the isogenic wild type in relation to streptonigrin resistance (lines 218-220); however, they concluded that there is a biological effect on the mutations. Thus, the importance of this finding is highly questionable.

**We agree with this comment, so we have removed this figure from the manuscript.**

8. In line 251, the authors state that the WT result is represented in figure 6, but it is not. As in point 1 above, the WT should be represented side- by-side with the pilE mutant to compare the magnitude of protection granted by the antioxidants against streptonigrin.

**We have made editorial changes to line 353 to omit the parental result to emphasize the focus on the *pilE* mutant.**

9. The result for the total iron content described in line 269 is not represented anywhere.

**Thank you, we have now included the results in the text in lines 380-382.**

10. In line 286-287 author discuss the effect of other ROS scavengers, but the result is not presented. Again, the data should be shown in a supplemental figure.

**We have made editorial changes to omit the ROS scavengers that had no effect.**

11. In lines 297-299 the explanation about the higher iron influx, which can fuel the Fenton reaction (and the consequent production of hydroxyl radicals and oxidative damage) in the pilE mutant seems to contradict the discussed result of figure S2 in which LL37 killing is not affected by addition of antioxidants.

**Our finding is that elevated iron in the *pilE* mutant increases sensitivity to LL-37 and hydrogen peroxide but only killing by hydrogen peroxide involves oxidative damage. Therefore, LL-37 sensitivity appears to involve iron but is ROS independent. We discuss better in the text in lines 394-395.**

12. In line 332 the authors suggest that “desferal could only access labile iron pool”. Do the authors know for sure that desferal can penetrate gonococci? If the authors have a reference in which it is shown that desferal makes it through the cell membrane, they should add it to the text.

**Unfortunately, there isn’t a reference for this but we have described streptonigrin as a hydrophobic molecule that is insoluble in water in lines 163-164. This implies that desferal is a membrane-diffusible compound.**

13. The authors should consider a summary figure that highlights the proposed mechanism of HP and LL-37 resistance with respect to pili and labile iron.

**We have included Figure 5 which describes the possible ways that pili can have these effects and described these hypotheses in lines 428-459.**

Major technical concerns:
14. The authors did not find any difference in total iron content by ICP-MS. Perhaps the difference between wild type and pilE mutant cells is where the iron is stored. If pilus bundles have some iron-chelation properties, then the wild type strain would accumulate iron at the cell surface or perhaps in the periplasm while the mutant would transport it directly into the cells. Have the authors considered performing the ICP-MS assay from gonococcal spheroplasts? Another alternative is to measure directly labile iron content using the colorimetric unified-ferene (u-ferene) assay, reported elsewhere. These experiments would help in supporting the conclusion that the way full piliation protect cells from hydrogen peroxide killing is by restricting the intracellular pool of labile iron.
15. Pyruvate can be a scavenger of toxic radicals produced by the Fenton reaction. Thus, the authors should consider testing the susceptibility of gonococci to HP and LL-37 when grown in pyruvate or by including pyruvate in the killing assays.

**Thank you for suggesting the unified-ferene assay. We have tried this assay but the results are not reproducible and will not be included in this manuscript**

**We did test pyruvate on LL-37 killing in the *pilE* mutant and did not observe a difference.**

1. Line 101 correct “predominantly”

**Corrected.**
2. Line 373 describe the concentration unit of glutamine

**Corrected.**
3. Line 373 double check concentration of Fe(NO3)3 in GC broth. In our GC broth recipe is 12.3 µM the final concentration.

**Corrected.**
4. Line 126 it should be panel D not C

**Corrected**
5. The syntax of sentence in line 132 is hard to understand

**We have changed the text in lines 161-162.**
6. Line 212 delete “for”

**This section of the text has been removed.**
7. Improve sentence writing in line 213. It is not easy to read

**This section of the text has been removed.**
8. Declare what the acronym of “ROS” stands for

**ROS is now described in line 72**
9. Line 245 add “the” after “through”

**Corrected..**
10. Line 250 change “then” to “when”

**Corrected.**
11. Line 292 change “with” to “by”

**Corrected**
12. Please clarify if N-1-60 is isogenic to FA1090.

**We have clarified this now in line 600 and it is in the strain table.**

Reviewer #2:

REPORT FOR TRANSMISSION TO AUTHORS

Hu Linda I. et al is a manuscript supporting evidence that the piliation state affects iron homeostasis in N. gonorrhoeae and influences the sensitivity to various antimicrobial agents, including hydrogen peroxide and the antimicrobial peptide LL-37. The topic is of interest to the field and is well done; The manuscript is excellent written, methods are robust and contain appropriate controls.

**We appreciate that the reviewer finds “the topic of interest to the field and is well done” and that the paper is written well.**

Figure 5: the authors should show data from three independent experiments for FA19.

**We have performed the repeats and the results are shown in Figure 3B.**

I would like to recommend to the authors to shorten the introduction and to focus better on the subject, which is dealt with in the manuscript. For example, the paragraph beginning at line 44 "N. gonorrhoeae primarily..." until line 54 could be deleted in its entirety.

**We believe that the information in the introduction is important for the reader, especially if they are new to this field. There are aspects of the symptoms of infection in men and women that we think are important to state. Additionally, this emphasizes the impact that gonorrhea can have on health, which is important to know.**

Minor comments:
- Page 6, line 91 and throughout the whole manuscript (e.g. line 198): type IV pili should be used consistently throughout the document or abbreviated

**Corrected**
- Figure 2: the authors should name the concentration of DMSO used or mention it in the material and methods section

**We have now included this information in lines 740-741 in the methods.**
- Figure 4: have the authors characterized the piliation status of the different mutants phenotypically (Immunoblotting, dot blot, EM)?

**This figure is no longer part of the revised manuscript.**
- Paragraph: Total iron content is unaffected by piliation : the authors mention that the parental strain and the pilE mutant showed no significant difference in total iron . data should be shown if possible (for example as Suppl figure S3)

**The data are now included in lines 380-382.**

Reviewer #3:

REPORT FOR TRANSMISSION TO AUTHORS

This report investigates the interesting observation that gonococcal pili protect against ROS and CAMPs by showing that there is a pilus-dependent effect on iron homeostasis. This is an interesting aspect of bacterial pathogenesis that is not well-explored and the data broaden the importance of the multi-functional type 4 pili in N. gonorrhoeae (Ng). Strengths of the manuscript include the straightforward description of experiments and the way the data are clearly shown in the figures.

**We appreciate that the reviewer also finds this observation interesting.**

The authors also test numerous mutants to explore their hypothesis. The results show that the process is likely multi- factorial, which makes defining the mechanism more difficult. This manuscript could be improved in terms of impact, however, if the authors at least provided a model that shows all the factors that might be involved and discussed each possible factor in more detail. Only one experiment is suggested (see below); the main issue is a need to at least help the reader visualize how the many factors hypothesized to play a role, based on the mutant testing, may do so, in absence of one or two easily tested mechanisms.

**Thank you for the suggestion to include a model. We have now done so in Figure 5.**

The authors should be commended for testing such a large number of mutants to explore their hypothesis. Results from the testing of pilus biogenesis genes and the complementation data with an inducible pilE gene support the hypothesis that the presence or assembled pili modulates intracellular iron. The conclusion that the effect of pili on streptonigrin killing is multifactorial also appears likely based on mutant data; however, more discussion of how these different is needed. For example, the results with the penicillin-binding protein mutants are interesting but more discussion would help the reader visualize what might be going on at the level of cell wall integrity or whatever the authors are thinking. No mention of the mpg gene is made after the introduction, and since Mpg also can affect PG, a more detailed discussion of the role of these three genes (dacB, dacC and mpG) in PG biosynthesis and/or degradation, and how their loss might lead to changes in iron homeostasis would increase the value of the data.

**Thank you. We decided that these mutants require further investigation and have removed the figure from the revised manuscript.**

Perhaps one possible mechanism could be tested: The authors state “Based on all the data presented, we propose that the differential sensitivity to streptonigrin, hydrogen peroxide, and LL-37 is due to piliation reducing the intracellular labile iron pool. This would be a possible explanation if piliation modulates iron import and, in the absence of the pilus, iron influx is uninhibited, leading to differential killing in the pilE mutant”. The authors showed that nonpiliated Ng do not have increased membrane permeability. Can the hypothesis that piliation modulates iron import be tested?

**We thank the reviewer for this suggestion. We believe that it is a next step for this research but is currently out of the scope of this paper.**

The authors mention pilin-dependent iron homeostasis (line 311). Some discussion about what is known about iron homeostasis in Ng is needed. Is there a known homeostasis system? Are there transcriptional regulators that alter this process? Is it known how ferrous iron enters the cell?

**We do not believe this speculative discussion would improve this manuscript.**

Numerous possibilities are listed in the discussion on page 17 as to how pilin or pilus may protect against ROS and elsewhere, how pili may directly or indirectly influence intracellular labile iron stores. A figure showing a model of all the possible forces that might lead to increased intracellular iron in nonpiliated Ng [– all of which are listed within the text in the discussion section (~lines 302 – 329)] - would help the reader think more about different mechanisms that contribute to this process (i.e. transcriptional networks (i.e. published data that are mentioned in the text), cell wall changes, direct or indirect of pilin on the electron transport chain (can this be tested?), efflux differences)

**We have included a model in Figure 5 and the accompanying text in the discussion lines 428-459.**

Page 4 “In men, infections can cause inflammation of the epididymis or epididymitis”. As written it sounds like epididymitis is a different condition than inflammation of the epididymis.

**We have changed the sentence in lines 50-51.**
Page 4: “These local gonococcal infections can also enter the bloodstream to develop disseminated gonococcal infection” The word “local” should be changed to “localized” or “mucosal”. Ascending infections (upper reproductive tract, male or female) aren’t actually considered localized (they are “locally disseminated” infections that can involve 2-3 organs, and so “mucosal” is the best adjective.

**Thank you. We have changed “local” to “mucosal” in line 52.**
Page 5 “Even in the presence of a bactericidal oxidative response when N. gonorrhoeae expressing outer membrane opacity (Opa) proteins variants can engage specific receptors on PMNs [25]… “This is a complicated sentence that may lose the reader who is not familiar with Ng Opa proteins/CEACAMS on neutrophils, etc.

**Thank you, we have edited the sentence in lines 75-77.**
Page 8 Vibrio cholera should be Vibrio cholerae

**Corrected.**
Page 34 “… determined using pSY6, a plasmid that carries a gyrA point mutant” - mutant should be mutation.

**Corrected.**
